# Root Fungal Endophytes Enhance Heavy-Metal Stress Tolerance of *Clethra barbinervis* Growing Naturally at Mining Sites via Growth Enhancement, Promotion of Nutrient Uptake and Decrease of Heavy-Metal Concentration

**DOI:** 10.1371/journal.pone.0169089

**Published:** 2016-12-28

**Authors:** Keiko Yamaji, Yumiko Watanabe, Hayato Masuya, Arisa Shigeto, Hiroshi Yui, Toshikatsu Haruma

**Affiliations:** 1 Graduate School of Life and Environmental Sciences, University of Tsukuba, Tsukuba, Ibaraki, Japan; 2 Tohoku Research Center, Forestry and Forest Products Research Institute, Morioka, Iwate, Japan; Estacion Experimental del Zaidin, SPAIN

## Abstract

*Clethra barbinervis* Sieb. et Zucc. is a tree species that grows naturally at several mine sites and seems to be tolerant of high concentrations of heavy metals, such as Cu, Zn, and Pb. The purpose of this study is to clarify the mechanism(s) underlying this species’ ability to tolerate the sites’ severe heavy-metal pollution by considering *C*. *barbinervis* interaction with root fungal endophytes. We measured the heavy metal concentrations of root-zone soil, leaves, branches, and fine roots collected from mature *C*. *barbinervis* at Hitachi mine. We isolated fungal endophytes from surface-sterilized root segments, and we examined the growth, and heavy metal and nutrient absorption of *C*. *barbinervis* seedlings growing in sterilized mine soil with or without root fungal endophytes. Field analyses showed that *C*. *barbinervis* contained considerably high amounts of Cu, Zn, and Pb in fine roots and Zn in leaves. The fungi, *Phialocephala fortinii*, *Rhizodermea veluwensis*, and *Rhizoscyphus* sp. were frequently isolated as dominant fungal endophyte species. Inoculation of these root fungal endophytes to *C*. *barbinervis* seedlings growing in sterilized mine soil indicated that these fungi significantly enhanced the growth of *C*. *barbinervis* seedlings, increased K uptake in shoots and reduced the concentrations of Cu, Ni, Zn, Cd, and Pb in roots. Without root fungal endophytes, *C*. *barbinervis* could hardly grow under the heavy-metal contaminated condition, showing chlorosis, a symptom of heavy-metal toxicity. Our results indicate that the tree *C*. *barbinervis* can tolerate high heavy-metal concentrations due to the support of root fungal endophytes including *P*. *fortinii*, *R*. *veluwensis*, and *Rhizoscyphus* sp. via growth enhancement, K uptake promotion and decrease of heavy metal concentrations.

## Introduction

Natural vegetation has been observed on the deposits containing high concentrations of heavy metals. These plant species must have evolved to adapt to the heavy-metal environment [1], because only plants showing heavy-metal tolerance can grow there. Today numerous pepperbush trees (*Clethra barbinervis* Sieb. et Zucc.) can be observed throughout the Hitachi mine forest, one of the three main copper mines of Japan in old days, although *C*. *barbinervis* was not among the species used for tree planting in the early 1900s [2]. The soil in Hitachi mine contains high concentrations of heavy metals, especially Cu, Ni, Zn, Cd and Pb [3,4], and many plant species suffer stunted growth there due to those soil conditions. *C*. *barbinervis* is a deciduous, broad-leaved, pioneer tree species, which grows naturally at mine sites [5]. *C*. *barbinervis* is also known to contain high concentrations of Zn, Mn, Co, Ni, and Cd in leaves; therefore, the leaves are used as a standard for heavy-metal analysis of natural products [6]. However, the heavy-metal tolerance mechanism(s) of *C*. *barbinervis* has yet to be clarified.

Some of microorganisms in the rhizosphere such as arbuscular mycorrhizal fungi, bacteria and fungi are known to enhance heavy-metal tolerance in plants. For example, arbuscular mycorrhizal fungi can accumulate heavy metals in the mycelia in order to inhibit the transfer of heavy metals to plant cells, resulting alleviation of their toxicity [7,8] (see also a review [9]). Ecological data suggest that arbuscular mycorrhizal fungi-infected plant species has increased in coal mine via a 28 year-investigation, indicating plant-growth enhancements by arbuscular mycorrhizal fungi under heavy-metal polluted site [10]. Rhizospheric bacteria are also known to decrease heavy metal toxicity in plants via enhancement of plant growth [11,12,13], producing siderophores and 1-aminocyclopropane-1-carboxylic acid (ACC) deaminase in the reduction of plant ethylene, for example. Recent studies have shown that root fungal endophytes can improve ecological adaptations of plants living in severe environments: root fungal endophytes can enhance the stress tolerance of plants to abiotic and biotic factors, including heat [14,15], salt [16,17], drought [18], herbivores [19,20], and pathogens [16,21,22] (see also reviews [23,24]). Root fungal endophytes such as dark-septate endophytes, induce tolerance of plants to heavy-metal stress, via enhancements of antioxidative system, changing heavy-metal distribution in plant cells and detoxification of heavy metal [25]. Thus, it is possible that microorganisms in the rhizosphere enhance stress tolerance in *C*. *barbinervis* growing under high concentrations of heavy metals.

The purpose of this study was to clarify the heavy-metal tolerance mechanism(s) of *C*. *barbinervis* by considering the species’ interaction with root fungal endophytes. Via field work (July 2006 to May 2007), we measured the heavy-metal concentrations of root-zone soil, and leaves, branches, and roots of mature *C*. *barbinervis* trees growing in the forest of the Hitachi mine. We isolated root fungal endophytes from surface-sterilized root segments in June, August, and October 2006. Through *in vitro* inoculation of root fungal endophytes to sterile *C*. *barbinervis* seedlings, we discerned whether root fungal endophytes are necessary for *C*. *barbinervis* to survive at sites with heavy-metal pollution.

## Materials and Methods

### Ethics statement

The study site belongs to Japanese National Forest. Our fieldwork activities, including observations and collections of plant materials and soil, were permitted by Ibaraki District Forest Office. Any endangered or protected species were not involved.

### Study site and sample collection

The study site is located in a forest containing *C*. *barbinervis* on the western slope (27.8°) of the Hitachi mine, Ibaraki prefecture, Japan (36°37′N, 140°38′E). Five *C*. *barbinervis* were arbitrarily selected; the average tree height ± SE was 15.0 ± 2.0 m and average tree age ± SE was 24.2 ± 3.9 years (assessed based on the number of annual rings extracted using an increment borer). These individuals were analyzed at monthly intervals for heavy-metal concentrations and root-endophyte isolation. The mean annual temperature during the study period (July 2006 to May 2007) was 14.6°C; mean temperature was highest in April 2006 (25.0°C) and lowest in January 2007 (6.1°C). Annual precipitation was about 1877 mm [26].

During the study period, root-zone soil, fine roots, leaves, and branches were collected for heavy-metal analysis from each *C*. *barbinervis* tree. Root-zone soil (50 × 50 × 50 mm) together with fine roots was collected within a 1-m diameter of each tree. After separating it from fine roots, root-zone soil was air-dried and passed through a 2-mm sieve mesh. According to the Fourth Committee for Unified Soil Classification System of Japan (Second Approximation) [27], our study site was considered to be forest brown soil. Leaves and branches of *C*. *barbinervis* were collected from 3-m height of each tree. Leaves were not collected from December 2006 to March 2007 (leaf buds of *C*. *barbinervis* grow in March and April, the leaves expand in April and May, turn yellow in October and November and fall in November and December). At each sampling, three leaves considered to be the same age were arbitrarily collected from each tree (*n* = 5) and combined to use for heavy-metal analysis.

### Chemical analysis of root-zone soil and plant materials

Total heavy metals (Cu, Ni, Zn, Cd, and Pb) in air-dried root-zone soil were quantified by inductively coupled plasma optical emission spectrometry (ICP-OES; model 757v, Nippon Jarrell-Ash, Kyoto, Japan) after digestion in concentrated HNO_3_–HClO_4_ (1:4 v/v). The results of five replications were averaged, and the standard errors were calculated.

Plant materials were carefully washed with running water and deionized water to remove soil particles according to a previous report [28]. These samples were separately dried for 48 h at 80°C and then ground with an electric mill (IFM-650D, Iwatani, Tokyo, Japan). After the ground materials were pyrolyzed in concentrated HNO_3_, their Cu, Ni, Zn, Cd, and Pb concentrations were quantified by ICP-OES. The results of five replications were averaged, and the standard errors were calculated.

### Root fungal endophyte isolation and microscopic observation of trypan-blue-stained roots

Fine roots were collected from five individual trees in June, April, and October 2006 and were used for root fungal endophyte isolation by means of the sterilization procedure described previously [29]. After fine roots were carefully washed with running water and deionized water, they were surface-sterilized with 70% ethanol for 1 min, followed by 15% hydrogen peroxide solution for 15 min and 70% ethanol for 1 min. The roots were then rinsed with sterilized deionized water to remove reagents, dried on sterile filter paper on a clean bench and then cut into approximately 5-mm pieces with a sterile scalpel. Thirty root pieces were randomly cut from individual roots of each *C*. *barbinervis*. The 150 root pieces were put on 1% malt extract agar (1% MA) and incubated at 23°C in the dark for 14 days. Fungal colonies were microscopically observed (100× and 400×, CX21, Olympus) and purified by means of serial plating on 1% MA. Root fungal endophyte detection rate (%) was calculated by the following formula:

Detectionrate(%)=NdNt×100

where *N*_d_ is the number of root pieces from which the fungus was detected and *N*_t_ is the total number of root pieces used for fungal isolation (150). Representative isolates used for inoculation were deposited in National Institute of Technology and Evaluation and the author’s (KY) private culture collection: *Phialocephala fortinii* C.J.K. Wang & H.E. Wilcox (NBRC111721), *Rhizodermea veluwensis* Verkley & Zijlstra (NBRC111722), and *Rhizoscyphus* sp. (D.J. Read) W.Y. Zhuang & Korf (NBRC111719).

The three dominant root-endophytic fungi isolated were identified based on morphological characteristics and molecular analysis [30]. For the molecular analysis, a small amount of mycelium was picked from the pure culture, crushed in 50 μl sterilized water, heated for 15 sec in a microwave oven, and 1-μl samples were used as templates for PCR. Fifty micro liters of PCR mixtures contained 25 μl of GoTaq master mix, 10 pmol of each primer for ITS regions (ITS5 and ITS4) [30], and deionized water. PCR was performed on a Bio-Rad T100 thermal cycler with the following protocol: an initial denaturing step at 94°C for 4 min; 35 cycles at 94°C for 30 sec, 52°C for 50 sec, and 72°C for 50 sec; and final elongation at 74°C for 6 min. Amplified samples were purified by a QIAquick PCR Purification Kit (Qiagen) and used for sequencing with a Big Dye Terminator Cycle Sequencing FS Ready Reaction kit ver. 3.1 on an ABI3100 genetic analyzer (Perkin-Elmer Applied Biosystems). Sequence data were deposited in the DNA Data Bank of Japan (DDBJ accession nos. LC151458-60). A sequence similarity search was performed for each series of obtained sequence data by using the BLAST program at the National Center for Biotechnology Information, and each taxon name was determined based on the result of the homology search. If identity according to the BLAST search was below 95% and a taxon name could not be determined, the sequence dataset was produced with the query sequence and the top 100 scoring sequences from the BLAST results. Phylogenetic trees (neighbor-joining) were produced from the dataset, and a taxon name was determined from each phylogenetic placement based on the clade.

To measure infection rates by arbuscular mycorrhizal fungi (*Paris* types; [31]) or fungal endophytes (microsclerotia; [32,33]), roots collected in October 2006 were stained with trypan blue and observed under the microscope [34]. Infection percentage of root length colonized was calculated according to the gridline-intersect method [35,36]. Results of five replications were averaged, and the standard errors were calculated.

### Inoculation test of *C*. *barbinervis* seedlings with root fungal endophytes

#### Preparation of sterile seedlings

Fallen seeds of *C*. *barbinervis* were collected at the study site in December 2006 and kept at 4°C before use. Seeds were soaked with deionized water in a vessel, and those that sank were used for the inoculation test. Seeds were dipped in 70% ethanol for 1 min, transferred into 15% hydrogen peroxide for 3 min, and dipped again in 70% ethanol for 1 min. Seeds were then rinsed with sterile deionized water and incubated on 3.5× diluted Hoagland medium containing 1.5% agar (light: 14 h, 25°C /dark: 10 h, 20°C) (Koitotoron, KOITO Manufacturing Co. Ltd.). Germination started after 2 weeks of incubation, and seedlings at the one true leaf stage were used for the inoculation test.

#### Properties of heavy-metal-polluted soil and sterilization

Root-zone soil collected in October 2006 was sealed within a plastic bag and sterilized by intermittent γ-irradiation (30 kGy). After sterilization, pH (H_2_O) and exchangeable Cu, Ni, Zn, Cd, and Pb were analyzed according to the methods described previously [37] to evaluate the γ-ray sterilization effect on chemical characteristics of soil. Exchangeable Cu, Ni, Zn, and Cd were extracted with 0.05 M Ca(NO_3_)_2_ (60 ml) from dried soil (6 g) by shaking at 150 rpm, 30°C, for 24 h. Exchangeable Pb was extracted with 1 M ammonium acetate (100 ml, pH 4.5) from 10 g dried soil by shaking at 150 rpm, 30°C, for 1 h. Results of three replications for pH (H_2_O) and five replications for exchangeable heavy metals were averaged, and the standard errors were calculated (S1 Table). On a clean bench, sterilized soil (350 ml) was transferred into a sterilized Agripot (Kirin) and sterile deionized water (30 ml) was added and used in the inoculation test.

#### Mycelial suspensions for the inoculation

Three dominant root fungal endophytes, *P*. *fortinii* (NBRC111721), *R*. *veluwensis* (NBRC111722), and *Rhizoscyphus* sp. (NBRC111719), were used for the inoculation test. These isolates were separately grown on 1% MA, and five mycelial disks (6-mm i.d.) on the edge of each mycelium were inoculated into a 100-ml Erlenmeyer flask containing 70 ml of 1% malt extract liquid medium. The medium was then statically incubated at 23°C in the dark for 3 weeks and then filtered through a sterile tea ball to obtain mycelia. The mycelia on the tea ball were fully rinsed with sterile deionized water to remove medium and then homogenized in sterile deionized water by a homogenizer (Nihonseiki, Ltd.) on ice for 3 min. Each mycelial suspension was prepared to contain 15 mg of mycelial dry weight (DW) per milliliter. Mixed mycelial suspension of the three root fungal endophytes (5 mg DW of each) was also prepared, autoclaved and used as a control, in order to account for the effect of nutrients supplied from dead mycelia on the seedling growth [38].

#### Inoculation test

Four sterile seedlings were aseptically transplanted to a pot containing sterilized rhizosphere soil, and each mycelial suspension (500 μl) of root fungal endophytes was inoculated close to the roots. The following mycelial suspensions were used for inoculation: (1) *P*. *fortinii*; (2) *R*. *veluwensis*; (3) *Rhizoscyphus* sp.; (4) mixed suspension of the three root fungal endophytes; and (5) autoclaved suspension (4) as a control. Three replicated pots were prepared per condition. The 12 seedlings per condition were grown for 40 days (light: 14 h, 25°C /dark: 10 h, 20°C; Koitotoron) and used for the following measurements: the number of leaves, height, and fresh weight (FW) of aboveground parts and roots. One seedling was randomly selected from each pot (i.e., three seedlings per condition) and used for the following measurements: root length, DW of aboveground parts and roots, and heavy metal and inorganic element concentrations. Plant materials were pyrolyzed in concentrated HNO_3_ and heavy metals (Cu, Ni, Zn, Cd, and Pb) and inorganic elements (P, Mg, Ca, K, Na, and Fe) were analyzed by ICP-OES. Replication results were averaged, and the standard errors were calculated.

After growth for 40 days, inoculants from roots were re-isolated and trypan-blue-stained roots were examined under the microscope to check whether this experiment was successful. For re-isolation, one seedling was randomly selected from each pot (i.e., three seedlings per condition). For microscopic observation of trypan-blue-stained roots, another seedling was randomly selected from each pot (four seedlings per condition).

### Fungal siderophore detection using the chrome azurol S assay

Fungal siderophores capable of chelating Fe and other heavy metals [39] were detected using a chrome azurol S (CAS) assay [40]. The root fungal endophytes *P*. *fortinii*, *R*. *veluwensis*, and *Rhizoscyphus* sp. were separately grown on 1% MA and five mycelial disks (8-mm i.d.) on the edge of each mycelium were inoculated into a 500-ml Erlenmeyer flask containing 400 ml of 1% malt extract liquid medium. The medium was incubated with shaking at 150 rpm at 23°C in the dark for 1 month. As a control, 1% MA agar disks were put into 1% malt extract liquid medium and the medium was incubated as described above. After incubation, each liquid medium was filtered through filter paper followed by a sterile 0.2-μm membrane filter (Advantec), and the filtrate was used to measure siderophore production by the CAS assay [40]. Siderophore concentration in the filtrate was quantified by the standard curve using deferoxamine mesylate (Calbiochem). Three replication results were averaged, and the standard errors were calculated.

### Statistical analysis

Statistical analysis was conducted by using version 21.0.0.0 of the SPSS statistics software for Macintosh (IBM). Differences of seedling growth variables, heavy metal concentrations, inorganic element concentrations or transfer factors of heavy metals between inoculation conditions were evaluated by one-factor ANOVA test (Tukey HSD). Differences were considered significant at *P* < 0.05.

## Results

### Heavy metal concentrations of root-zone soil, leaves, branches, and fine roots of *C*. *barbinervis* at our study site

In root-zone soil, Cu, Zn, and Pb concentrations were high throughout the year and Zn slightly showed seasonal variation (S1 Fig); the Zn concentration was highest in December, when the fallen leaves were supplied to the soil. Fine roots and branches contained higher Cu, Zn and Pb concentrations throughout the sampling period than Ni and Cd. No strong seasonal variations of heavy-metal concentrations were found in branches and fine roots (S1 Fig). In leaves, the Zn concentration was markedly higher than those of other heavy metals throughout the sampling period. Zn showed a seasonal variation in leaves; the Zn concentration was highest in November, when the leaves turn yellow (S1 Fig). Among transfer factors (ratios of root concentration to root-zone soil concentration) of heavy metals (S2 Table), those of Zn were highest in leaves and branches. In fine roots, the transfer factor of Cd was highest, although the Cd concentration was not high (S1 Fig).

### Root fungal endophyte isolation and fungal infections inside roots

Root fungal endophytes were isolated from fine roots of mature *C*. *barbinervis* trees, and the detection rates are shown in Table 1. Throughout the sampling period, three fungal endophyte species were dominant, and DNA analyses clarified that those fungal endophytes were *P*. *fortinii*, *R*. *veluwensis*, and *Rhizoscyphus* sp. Microscopic observation of trypan-blue-stained fine roots collected in October 2006 revealed that infection rates by arbuscular mycorrhizal fungi (mainly *Paris* types) and fungal endophytes (microsclerotia) were 53.8 ± 2.7% and 39.6 ± 2.7%, respectively.

**Table 1:** Detection rates of fungi isolated from *C*. *barbinervis* roots.

Fungus	June (%)	August (%)	October (%)	Mean±SE (%)
*Phialocephala fortinii*	5	4	14	7.7 ± 3.2
*Rhizodermea veluwensis*	11	10	22	14.2 ± 3.8
*Rhizoscyphus* sp.	25	34	23	26.8 ± 3.4

The means of percentages in June, August, and October are shown with ±SE.

### Effect of root fungal endophytes on *C*. *barbinervis* seedling growth and concentrations of inorganic elements and heavy metals in the inoculation test

In the inoculation test, we used three root endophytes, *P*. *fortinii*, *R*. *veluwensis*, and *Rhizoscyphus* sp., that were isolated from mature *C*. *barbinervis* trees growing at the mine site. After 40 days of incubation, we considered that our inoculation test was successful without contamination, because all root fungal endophytes were isolated from roots inoculated with root fungal endophytes and no microorganisms were isolated from control roots. Our incubation time was apparently sufficient for fungal endophytes to interact with roots, because the infection rates of *P*. *fortinii*, *R*. *veluwensis*, *Rhizoscyphus* sp., and mixed inoculation of the three species were 81.3 ± 2.5%, 82.9 ± 5.5%, 77.8 ± 4.7%, and 65.6 ± 4.8%, respectively.

**Fig 1:**
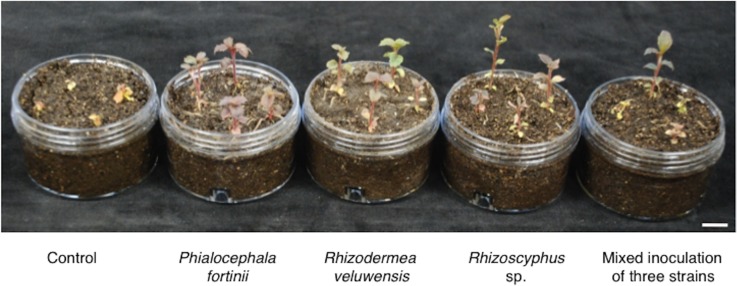
Root fungal endophyte inoculation increased *C*. *barbinervis* seedling growth in the inoculation test. Seedlings were grown for 40 days in sterilized heavy-metal-polluted soil. Scale bar represents 10 mm.

Root fungal endophyte inoculation increased *C*. *barbinervis* seedling growth in heavy-metal polluted soil (Fig 1, Table 2). In particular, *P*. *fortinii* and *Rhizoscyphus* sp. significantly increased the number of *C*. *barbinervis* leaves, height, root length, and root FW compared with those parameters in the control (*P* < 0.05). Additionally, *P*. *fortinii* significantly increased aboveground part FW and *Rhizoscyphus* sp. significantly increased root DW. *Rhizodermea veluwensis* significantly increased only the number of leaves (*P* < 0.05), but showed a tendency to increase growth parameters such as height (*P* = 0.116) and aboveground part DW (*P* = 0.249). Mixed inoculation of the three strains also showed a tendency to increase the number of leaves (*P* = 0.059), height (*P* = 0.138), and aboveground part FW (*P* = 0.230). In contrast, the growth of control seedlings was poor, with leaf chlorosis and inhibition of root tip growth.

**Table 2:** Seedling growth parameters after the inoculation test.

Treatment	Number of leaves	Height (mm)	Root length (cm)	Aboveground part FW (mg)	Root FW (mg)	Aboveground part DW (mg)	Root DW (mg)
Control	5.0 ± 0.3 a	5.9 ± 0.4 a	10.8 ± 2.9 a	5.8 ± 1.1 a	1.8 ± 0.2 a	5.1 ± 1.3 a	0.8 ± 0.0 a
*Phialocephala fortinii*	11.1 ± 0.9 b	28.1 ± 3.7 c	123.0 ± 23.4 b	61.4 ± 10.8 b	49.2 ± 9.3 c	23.0 ± 4.0 a	10.7 ± 1.37 ab
*Rhizodermea veluwensis*	10.9 ± 1.6 b	15.7 ± 3.0 ab	71.6 ± 22.7 ab	30.0 ± 7.6 ab	11.7 ± 3.1 ab	19.7 ± 5.1 a	5.5 ± 1.4 ab
*Rhizoscyphus* sp.	10.6 ± 1.4 b	18.5 ± 3.3 bc	134.5 ± 37.1 b	39.6 ± 11.8 ab	34.1 ± 12.5 bc	23.7 ± 7.9 a	11.2 ± 4.1 b
Mixture	9.3 ± 0.6 ab	15.3 ± 2.3 ab	71.5 ± 10.4 ab	32.4 ± 9.1 ab	21.6 ± 7.6 abc	13.0 ± 1.5 a	6.5 ± 1.6 ab

FW: fresh weight. DW: dry weight. Different letters indicate a statistically significant difference among treatments in ANOVA comparisons and post-hoc Tukey HSD at *P* < 0.05. For number of leaves, height, shoot FW, and root FW, *n* = 12. For root length, shoot DW, and root DW, *n* = 3. The means are shown with ±SE.

**Fig 2:**
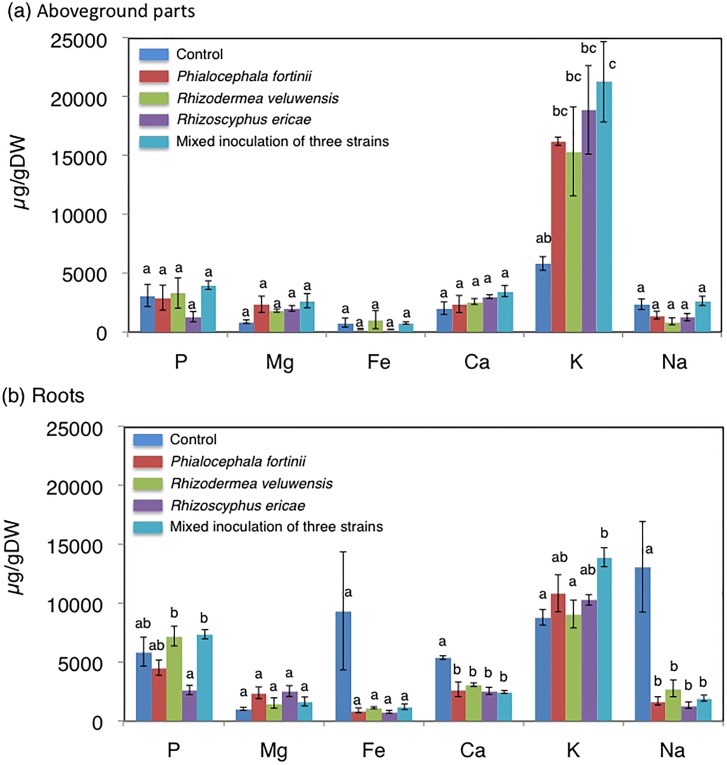
Nutrient element concentrations in *C*. *barbinervis* seedlings in the inoculation test. (a) Concentrations in aboveground parts, and (b) concentrations in roots. Different letters indicate a statistically significant difference among treatments in ANOVA comparisons and the post-hoc Tukey HSD at *P* < 0.05. Error bars represent ± SE.

**Fig 3:**
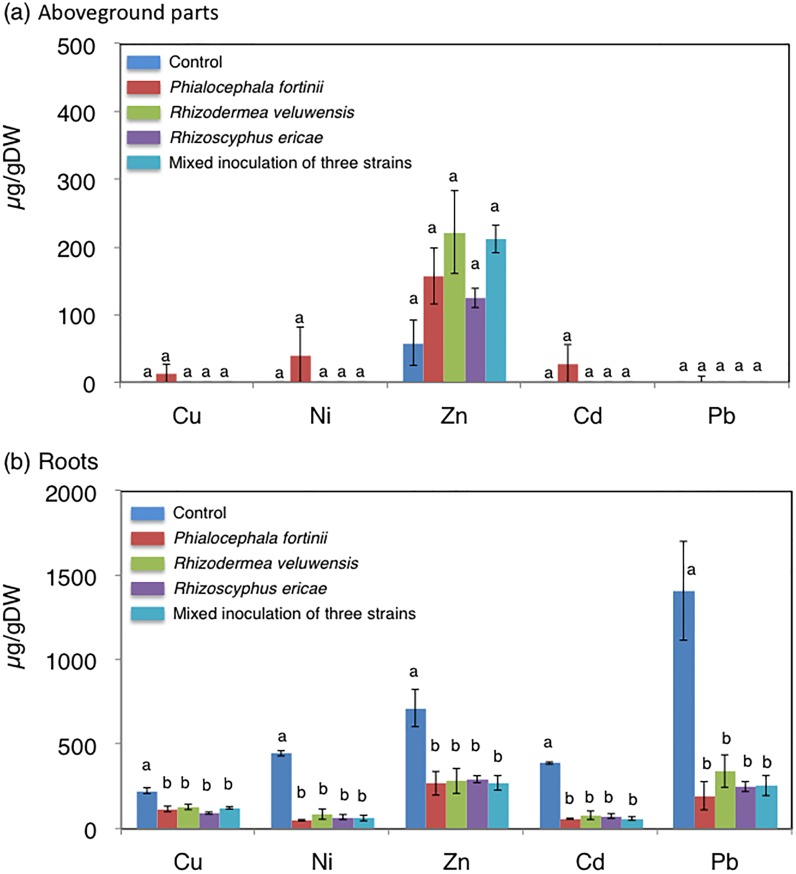
Heavy-metal concentrations in *C*. *barbinervis* seedlings in the inoculation test. (a) Concentrations in aboveground parts, and (b) concentrations in roots. Different letters indicate a statistically significant difference among treatments in ANOVA comparisons and the post-hoc Tukey HSD at *P* < 0.05. Error bars represent ± SE.

After incubation, concentrations of plant nutrients (P, Mg, K, Na, and Fe) were analyzed in aboveground parts (Fig 2A) and roots (Fig 2B). Concentrations of heavy metals (Cu, Ni, Zn, Cd, and Pb), which were higher in root-zone soil than in unpolluted soil (S2 Table) [3,4], were also analyzed in aboveground parts (Fig 3A) and roots (Fig 3B). In the aboveground parts, compared with the control, K concentration was increased significantly by mixed inoculation of the three strains (*P* < 0.05), and slightly by *P*. *fortinii* (*P* = 0.149), *R*. *veluwensis* (*P* = 0.203), and *Rhizoscyphus* sp. (*P* = 0.054; Fig 2A). In contrast, the secondary macronutrient Ca and beneficial nutrient Na were significantly decreased in roots by all four inoculation conditions (*P* < 0.05; Fig 2B). All heavy metals (Fig 3B) were also decreased in the roots by all four inoculation conditions (*P* < 0.05). The transfer factors shown in Table 3 indicate that all heavy metals were transferred at a greater rate to control roots as compared with fungal endophyte-inoculated roots.

**Table 3:** Transfer factors (ratios of root concentration to soil concentration) of heavy metals in the inoculation test.

Treatment	Transfer factor Cu	Transfer factor Ni	Transfer factor Zn	Transfer factor Cd	Transfer factor Pb
Control	0.44 ± 0.03 a	33.7 ± 0.04 a	4.38 ± 0.67 a	59.0 ± 1.03 a	2.32 ± 0.48 a
*Phialocephala fortinii*	0.23 ± 0.03 b	3.82 ± 0.03 b	1.65 ± 0.43 b	8.99 ± 0.48 b	0.32 ± 0.14 b
*Rhizodermea veluwensis*	0.25 ± 0.03 b	6.53 ± 2.32 b	1.74 ± 0.45 b	12.1 ± 3.85 b	0.56 ± 0.16 b
*Rhizoscyphus* sp.	0.18 ± 0.01 b	5.25 ± 1.11 b	1.80 ± 0.13 b	11.5 ± 2.14 b	0.41 ± 0.05 b
Mixture	0.24 ± 0.01 b	4.78 ± 1.25 b	1.67 ± 0.27 b	9.15 ± 1.59 b	0.42 ± 0.10 b

Transfer factor (ratio of root concentration to soil concentration used for the inoculation test) was calculated using each sample. The means are shown with ±SE. Different letters indicate a statistically significant difference among treatments in ANOVA comparisons and post-hoc Tukey HSD at *P* < 0.05. *n* = 3.

### Siderophore production by root fungal endophytes

*Phialocephala fortinii* and *R*. *veluwensis* produced siderophores, whereas *Rhizoscyphus* sp. did not. Siderophore concentrations in the liquid medium of *P*. *fortinii* and *R*. *veluwensis* were 2.93 ± 1.46 μM and 13.0 ± 2.37 μM, respectively.

## Discussion

Because *C*. *barbinervis* grows in natural habitats at many mining sites [5], including the Hitachi mine, *C*. *barbinervis* is considered to be a heavy-metal-tolerant tree species, similar to metal-hypertolerant species, which can survive and reproduce on highly metal-enriched soils [41,42]. Concentrations of heavy metals (Cu, Zn, and Pb) were considerably high, especially in the fine roots of mature trees of *C*. *barbinervis* growing at our study site (S1 Fig), compared with previously reported critical toxicity levels in land plants (Cu, 20–30 μg/g; Zn, 100–300 μg/g; Pb, 0.6–28 μg/g) [43]. The concentrations measured in this study were also within the ranges of Cu, Zn, and Pb concentrations in plants grown at contaminated sites (Cu, 2–1123 μg/g; Zn, 21–2600 μg/g; Pb, 32–1506 μg/g) [44], even though the translocation of heavy metals from soil to plant tissues was not high (S2 Table). However, without root fungal endophytes, *C*. *barbinervis* seedlings could not grow well in soil from the site (Fig 1, Table 1), indicating that the heavy-metal tolerance of *C*. *barbinervis* was conferred by the presence of root fungal endophytes.

Sites polluted with heavy metals are severe environments for the growth of both plants and microbes. Root fungal endophytes show “weak harm or benefit” to plants, which means that they can be weak pathogens or symbionts [45] according to their growing environments. Under stressful environments such as heavy-metal polluted sites, where plants and microbes need to survive together, the interactions between plants and root fungal endophytes would be beneficial to both [46]. Our inoculation test using the dominant root fungal endophytes isolated from *C*. *barbinervis* roots (Table 1), clarified that *P*. *fortinii*, *R*. *veluwensis*, and *Rhizoscyphus* sp. are symbionts under heavy-metal stress conditions. Root fungal endophytes could enhance their own heavy-metal tolerance via extracellular (chelation, cell-wall binding) and/or intracellular (binding to detoxicants, compartmentation) detoxification mechanisms [47,48]. For example, dark septate root endophytes (DSEs) like *P*. *fortinii* can produce the black biopolymer melanin, which can be synthesized from phenolics and binds heavy metals [49,50]. In turn, heavy-metal binding to melanin (compartmentation in fungal cell wall) would enable heavy-metal ions to be kept away from living plant cells [51].

As shown in the inoculation test (Figs 1 and 2, Table 2), growth enhancement and promotion of uptake of the essential macronutrient K by root fungal endophytes are also effective means of promoting heavy-metal tolerance in plant tissues: when plants grow extremely quickly, concentrations of inorganic nutrients in plants decrease (dilution effect) without keeping up nutrient absorption [1]. Thus, dilution of heavy metals in plant tissues resulting from rapid plant growth may decrease the toxicity of heavy metals [52]. This suggestion was also supported by the low transfer ratios of heavy metals from root-zone soil to roots inoculated with root fungal endophytes (Table 3).

Siderophores, which are metal-chelating compounds [53,54], would be helpful in inhibiting absorption of heavy metals into plant cells because siderophores released from roots into the rhizosphere can form complexes with heavy metals that are not easily absorbed by plant roots. In our study, *P*. *fortinii* and *R*. *veluwensis* showed an ability to produce siderophores, and their siderophore production probably affects heavy-metal exclusion in the rhizosphere. However, *Rhizoscyphus* sp., which also enhanced heavy-metal tolerance in our inoculation test, did not show siderophore production abilities. In that case, there must be multiple mechanisms that enhance heavy-metal tolerance in *C*. *barbinervis*.

DSE fungi are reported to improve heavy-metal tolerance in plants such as maize [55]; DSE isolates from lead and zinc mining and smelting sites show high heavy-metal tolerance and accumulate heavy metals in their mycelia, promising the enhancement of heavy-metal tolerance in plants as well [56]. Compared with DSEs such as those in the genus *Phialocephala*, *Rhizodermea* and *Rhizoscyphus* are nonclavicipitaceous endophytes, which broadly and extensively colonize shoots, roots, and rhizomes and some of which confer drought, heat, and pathogen tolerance in plants [24]. However, except for our study, *Rhizodermea* and *Rhizoscyphus* have not been reported to symbiotically interact with plants growing under heavy-metal stress. For example, *Rhizoscyphus ericae* is an ericoid mycorrhizal fungus that infects leafy liverworts or ectomycorrhizal and ericoid mycorrhizal tree roots [57,58]. *Rhizodermea veluwensis* is a recently described species that was isolated from surface-sterilized roots of *Erica tetralix*, *Empetrum nigrum*, and *Vaccinium* spp., plants of the family Ericaceae, and also *Larix decidua* [59]. It belongs in Dermateaceae, Helotiales, Leotiomycetes, Ascomycota, but its teleomorph was not found. DSEs can promote nitrogen uptake in plant cells [33,60]. We were unable to analyze nitrogen uptake enhancement by root fungal endophytes due to an insufficient number of plant samples in our inoculation test. The ecological roles of root fungal endophytes such as DSEs have been studied to a limited extent [23], and research conducted in natural ecosystems should clarify whether root fungal endophytes protect their hosts from environmental stresses.

Arbuscular mycorrhizal fungi that infect *C*. *barbinervis* roots [61] are also known to live in a symbiotic relationship with approximately 80–90% of terrestrial plants [62]. They increase heavy-metal tolerance of plants via reduction of heavy-metal uptake into plant cells (see review [63]), via secretion of glomalin, which can bind heavy metals to exclude them from roots [64], or via heavy-metal accumulation in the fungal cell wall and vesicles [65]. Generally, arbuscular mycorrhizal fungi and root fungal endophytes coexist in plant roots [66,67]. Even though arbuscular mycorrhizal fungi are known to be weak in heavy-metal tolerance compared with DSEs [68] in heavy-metal-polluted areas such as our study site, in future research we should consider the simultaneous effect of both fungal species on the enhancement of heavy-metal tolerance in plants.

## Conclusions

We identified one reason why *C*. *barbinervis* can survive in heavy-metal-polluted soils such as mining sites: root fungal endophytes can enhance the heavy-metal tolerance of *C*. *barbinervis* via growth enhancement, K uptake and decrease of heavy-metal concentration in plant cells. *C*. *barbinervis* is not a hyperaccumulator of heavy metals and is not used for phytoextraction. However, it is useful for phytostabilization, which decreases heavy-metal bioavailability in soil via precipitation of heavy metals into less soluble forms by plant and microbial metabolites or accumulation in root tissues [69], resulting in the inhibition of heavy-metal dispersion outside a polluted site. Mine sites, where heavy-metal removal is very difficult because of extremely high concentrations, are suitable areas for tree planting as part of phytostabilization. Furthermore, root-endophyte inoculation would improve the success of rapid seedling establishment at mine sites. In those cases, the plant and microbial species used should not be non-native, in order to maintain regional plant diversity [69]. Our research suggests that the selection of tolerant plants together with their supporting microbes from among native species is the most appropriate way to perform phytostabilization.

## Supporting Information

S1 Fig Heavy metal concentrations in root-zone soil and *C*. *barbinervis* organs during the sampling period. (a) Root-zone soil. (b) Fine roots. (c) Branches. (d) Leaves. Error bars represent ± SE.
(TIF)

S1 Table pH (H_2_O) and exchangeable heavy metals (mg/kg DW) in non-sterile and γ-ray sterilized soils. Results are expressed as average ± SE.
(DOCX)

S2 Table Transfer factors (ratios of concentration in plant organs to soil concentration) of heavy metals in *C*. *barbinervis*. Transfer factor (ratio of leaf or branch or root concentration to root-zone soil concentration) was calculated using each sample during the sampling period. The means are shown with ±SE.
(DOCX)
