# Supplementary figures and images for: Root Fungal Endophytes Enhance Heavy-Metal Stress Tolerance of Clethra barbinervis Growing Naturally at Mining Sites via Growth Enhancement, Promotion of Nutrient Uptake and Decrease of Heavy-Metal Concentration
Source: PLoS One. 2016 Dec 28;11(12):e0169089. doi: 10.1371/journal.pone.0169089 (PMC5193448; doi:10.1371/journal.pone.0169089)

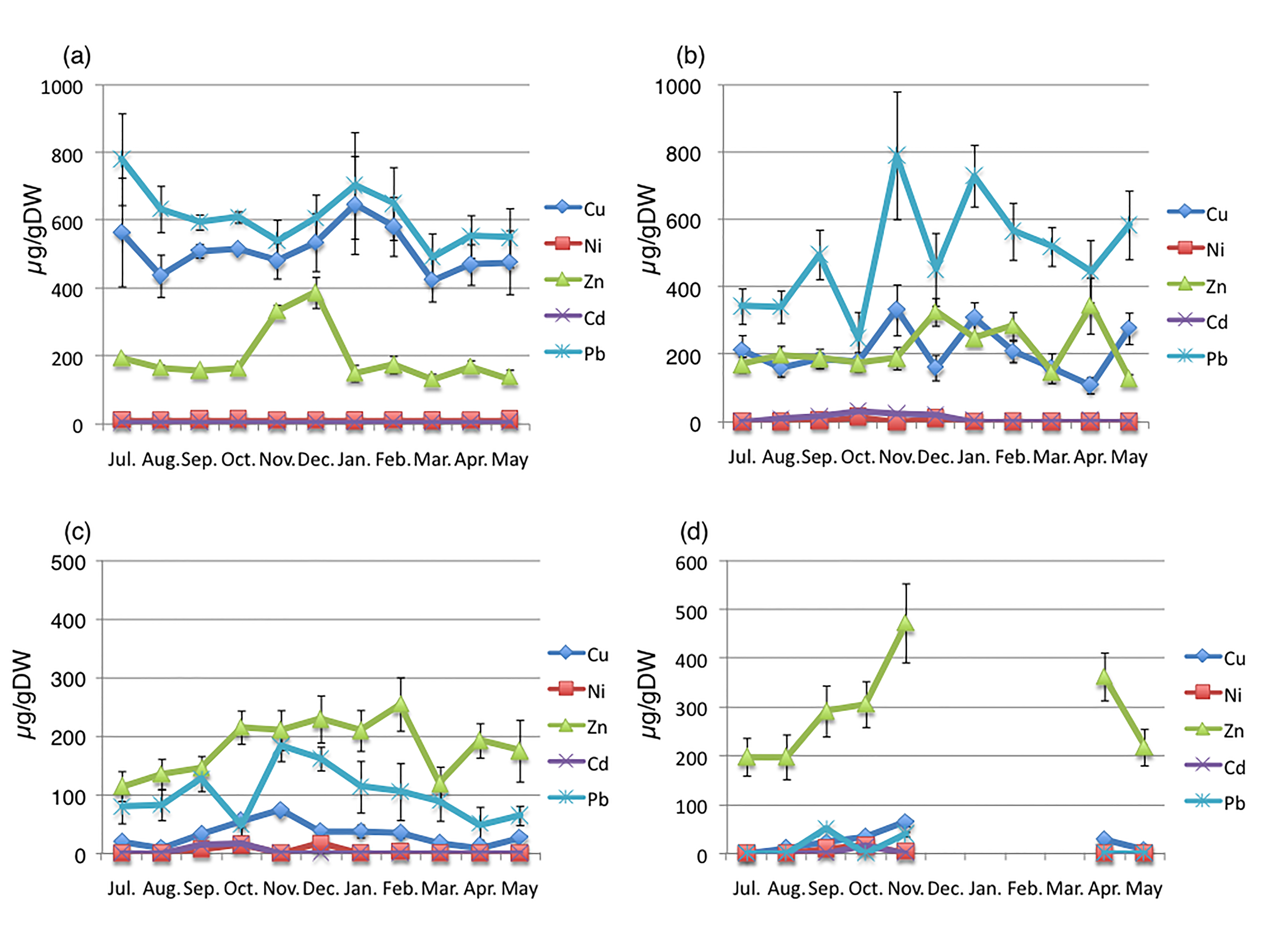

Supplement: S1 Fig — (a) Root-zone soil. (b) Fine roots. (c) Branches. (d) Leaves. Error bars represent ± SE. (TIF) [file pone.0169089.s001.tif]
