# Supplementary material for: Root Fungal Endophytes Enhance Heavy-Metal Stress Tolerance of Clethra barbinervis Growing Naturally at Mining Sites via Growth Enhancement, Promotion of Nutrient Uptake and Decrease of Heavy-Metal Concentration
Source: PLoS One. 2016 Dec 28;11(12):e0169089. doi: 10.1371/journal.pone.0169089 (PMC5193448; doi:10.1371/journal.pone.0169089)
Supplement: S1 Table — Results are expressed as average ± SE. (DOCX) [file pone.0169089.s002.docx]

S1 Table. pH (H_2_O) and exchangeable heavy metals (mg/kg DW) in non-sterile and γ-ray sterilized soils.

|  | Non-sterile soil | Sterile soil by γ-ray |
| --- | --- | --- |
| pH (H_2_O) | 3.50 ± 0.00 | 3.60 ± 0.10 |
| Exchangeable Cu | 3.34 ± 0.11 | 3.82 ± 0.05 |
| Exchangeable Ni | 2.42 ± 0.03 | 2.38 ± 0.05 |
| Exchangeable Zn | 68.5 ± 0.23 | 71.4 ± 0.20 |
| Exchangeable Cd | 1.63 ± 0.02 | 1.55 ± 0.01 |
| Exchangeable Pb | 58.3 ± 0.17 | 58.7 ± 0.26 |

Results are expressed as average ± SE.
