# Supplementary material for: Root Fungal Endophytes Enhance Heavy-Metal Stress Tolerance of Clethra barbinervis Growing Naturally at Mining Sites via Growth Enhancement, Promotion of Nutrient Uptake and Decrease of Heavy-Metal Concentration
Source: PLoS One. 2016 Dec 28;11(12):e0169089. doi: 10.1371/journal.pone.0169089 (PMC5193448; doi:10.1371/journal.pone.0169089)
Supplement: S2 Table — Transfer factor (ratio of leaf or branch or root concentration to root-zone soil concentration) was calculated using each sample during the sampling period. The means are shown with ±SE. (DOCX) [file pone.0169089.s003.docx]

S2 Table. Transfer factors (ratios of concentration in plant organ to soil concentration) of heavy metals in *C. barvinervis.*

| Samples | Cu | Ni | Zn | Cd | Pb |
| --- | --- | --- | --- | --- | --- |
| Leaves | 0.05 ± 0.01 | 0.32 ± 0.08 | 1.74 ± 0.14 | 0.33 ± 0.13 | 0.02 ± 0.01 |
| Branches | 0.07 ± 0.01 | 0.33 ± 0.08 | 1.04 ± 0.08 | 0.26 ± 0.10 | 0.18 ± 0.02 |
| Fine roots | 0.41 ± 0.03 | 0.22 ±0.06 | 1.24 ± 0.09 | 2.19 ± 0.42 | 0.89 ± 0.07 |

Transfer factor (ratio of leaf or branch or root concentration to root-zone soil concentration) was

calculated using each sample during the sampling period. The means are shown with ±SE.
